# Aripiprazole Therapy During Pregnancy: Case Report and Clinical Considerations for Maternal and Fetal Health

**DOI:** 10.1192/j.eurpsy.2026.11936

**Published:** 2026-07-31

**Authors:** A. Pirtovšek Šavs, M. Derganc

**Affiliations:** University Psychiatric Clinic Ljubljana, Ljubljana, Slovenia

## Abstract

**Introduction:**

The management of schizophrenia during pregnancy remains a significant clinical challenge. Only a limited number of antipsychotics are considered relatively safe for use, and even these are not appropriate for all patients. Clinicians must carefully balance maternal psychiatric stability with fetal safety, while considering the risks of untreated psychosis, medication side effects, and relapse in the perinatal period. Reports on long-term outcomes with specific agents remain scarce, particularly for newer antipsychotics such as aripiprazole.

**Objectives:**

Case Presentation:

We present the case of a 28-year-old woman, a microbiologist by profession, who was admitted to the University Psychiatric Hospital Ljubljana during her second psychotic episode. Clinical features included persecutory delusions, perceptual disturbances, and disorganized behavior, fulfilling criteria for paranoid schizophrenia. During her admission, she initially received risperidone alongside aripiprazole, but treatment was gradually optimized to aripiprazole monotherapy. On a dose of 20 mg daily she achieved complete remission, with marked improvement in motivation and functioning, and with minimal adverse effects. Maintenance therapy was subsequently continued at 10 mg daily. Eighteen months later, while stable on this regimen, the patient conceived. She remained psychiatrically stable throughout pregnancy, and although delivery occurred one month prematurely, both mother and infant were healthy, and the postpartum course was uneventful. Importantly, she experienced no psychotic relapse. Two years later she conceived again, while still on aripiprazole 10 mg. The second pregnancy was uncomplicated, and she delivered a healthy girl. Again, no psychiatric decompensation was observed during pregnancy or postpartum, and maternal functioning was preserved.

**Conclusions:**

This case highlights that continuous treatment with aripiprazole can support sustained remission of schizophrenia during pregnancy and postpartum without obvious adverse consequences for mother or child. While single cases must be interpreted with caution, the report adds to emerging clinical experience suggesting that aripiprazole may represent a viable therapeutic option for pregnant women with schizophrenia. Its relative tolerability, maintenance of remission, and lack of observed perinatal complications in this case underscore the need for further systematic research into its safety and effectiveness in this vulnerable population.

**Disclosure of Interest:**

None Declared

